# Multimodal Sentiment Analysis Representations Learning via Contrastive Learning with Condense Attention Fusion

**DOI:** 10.3390/s23052679

**Published:** 2023-03-01

**Authors:** Huiru Wang, Xiuhong Li, Zenyu Ren, Min Wang, Chunming Ma

**Affiliations:** 1 Xinjiang Key Laboratory of Signal Detection and Processing, College of Information Science and Engineering, Xinjiang University, Urumqi 830046, China; 2 College of Information Science and Engineering, Xinjiang University, Urumqi 830046, China

**Keywords:** multimodal, multimodal sentiment analysis, supervised contrastive learning, MLFC, SCSupCon

## Abstract

Multimodal sentiment analysis has gained popularity as a research field for its ability to predict users’ emotional tendencies more comprehensively. The data fusion module is a critical component of multimodal sentiment analysis, as it allows for integrating information from multiple modalities. However, it is challenging to combine modalities and remove redundant information effectively. In our research, we address these challenges by proposing a multimodal sentiment analysis model based on supervised contrastive learning, which leads to more effective data representation and richer multimodal features. Specifically, we introduce the MLFC module, which utilizes a convolutional neural network (CNN) and Transformer to solve the redundancy problem of each modal feature and reduce irrelevant information. Moreover, our model employs supervised contrastive learning to enhance its ability to learn standard sentiment features from data. We evaluate our model on three widely-used datasets, namely MVSA-single, MVSA-multiple, and HFM, demonstrating that our model outperforms the state-of-the-art model. Finally, we conduct ablation experiments to validate the efficacy of our proposed method.

## 1. Introduction

With the rapid development of the Internet, multimodal social media sites are becoming increasingly popular. Users increasingly use data in multiple modalities to express their emotions, the most common of which is a combination of text and images. Social media sentiment analysis can analyze people’s attitudes toward hot topics and important events, and it has a high research value in box office prediction and product recommendations. As a result, multimodal sentiment analysis based on text and images has emerged as a research focus in recent years [1]. Early research focused on sentiment analysis on single-modal data (images, text, or video). Text sentiment analysis refers to the task of mining through the emotional polarity contained in a given text [2]. Text sentiment analysis using deep learning methods usually integrates multiple low-level machine learning models, uses neural networks to encode the text contextually, and converts low-level vector representations into high-level vector representations [3]. This method has achieved good results in sentence-level and chapter-level sentiment analysis tasks. In [4], they proposed the Interactive Attention Network (IAN), which enables interactive learning of target and contextual attention. In [5], they proposed a multi-attention network (MAN), which solves the problem of information loss by using intralayer and interlayer attention mechanisms for aspect-based sentiment analysis. Visual sentiment analysis uses image processing techniques to extract and recognize emotions from facial expressions and body gestures. The literature [6,7] has achieved good results in sentiment analysis of large-scale visual content. Although sentiment analysis for single-modal data has been applied to product recommendation and consumption prediction, there are still shortcomings, such as low recognition rate and poor generalization, due to factors such as single expression form and insufficient information. Multiple modalities’ data frequently have a certain complementarity, which can compensate for each other’s shortcomings. The data in the sentiment analysis task of large-scale visual content in the literature lacks complementary modal information, making the model’s representation more incomplete.

**Figure 1:**
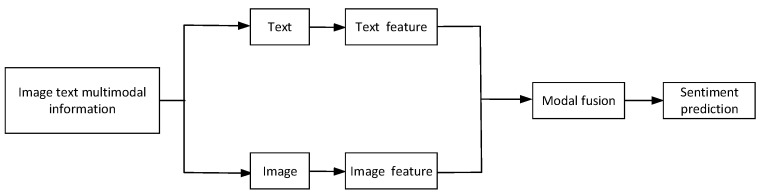
Illustration of Fusion method. The image and text are extracted by features for the multimodal information of image text corresponding to the model input, and the emotional polarity is judged after feature fusion.

In recent years, sentiment analysis research has been different from previous ones, focusing on using deep learning-related techniques and multiple modalities to improve results in sentiment analysis tasks [8,9,10]. After entering multimodal data, extract the characteristics of pictures and texts, obtain the representation information of each modality, and then select the appropriate multimodal data fusion algorithm to fuse various modal information to obtain multimodal representation. Based on this representation information, sentiment analysis is performed. Figure 1 depicts the primary flow.

You et al. proposed cross-modality consistent regression. This CCR model, for the first time, constructed a semantic tree structure based on sentence resolution to align areas of text and images for accurate analysis [11]. In 2017, Chen et al. explored an end-to-end deep fusion convolutional neural network for co-learning text and visual emotional representations, effectively fusing bimodal information of text and images and predicting overall sentiment [12]. Tree-structured Recursive Neural Networks were proposed by [13]. Construct a semantic tree structure based on sentence parsing and fused with visual attention. In [14], they proposed a novel multimodal emotion analysis model based on the Multi-view Attentional Network (MVAN) that employs a memory network to achieve information interaction between modalities and obtain deep semantic features of images and text with promising results.

In multimodal sentiment analysis, there are three main challenges. First, it faces problems related to model performance, such as training speed, inference speed, and model parameters. Because multimodal models need to model multiple modalities, the model volume becomes more significant, and performance suffers. Second, there needs to be more labeling data resources. At present, the primary multimodal datasets used are the Flickr30k dataset [15], the VQA dataset [16], and the CMU-MOSEI dataset [17]. Moreover, a model trained in one specific scenario cannot be directly applied to other scenarios.

This paper mainly analyzes sentiment information in two modes: pictures and text. Unlike single-modality models, multimodal models can extract better and more complete feature representations and achieve the desired effect after fusion by utilizing the association information between each modality. Contrastive learning has recently gained popularity because it solves the problem of less or no labeling, constructs similar samples as positive samples through data enhancement, and can learn more common features for sentiment analysis tasks. The CLMLF model employs contrastive learning to learn sentimental representations containing multimodal data. However, due to the small number of positive samples and the presence of false positive samples, we implemented a supervised comparative learning task and added loss to bring the same type of sample as close as possible and different types of samples as far away as possible. Our task entails avoiding the problem of misclassified negative samples in supervised sample situations and improving classification problem accuracy, which is ideal for contrastive learning. Based on the earlier challenges, this paper proposes a multimodal sentiment analysis approach incorporating CNNs in a fusion approach to merge the original Transformer structure. We compare the model’s effectiveness to all baseline models on the MVSA-Single, MVSA-Multiple [18], and HFM datasets [19]. The experimental results show that our proposed model has a better detection effect. A comprehensive set of ablation experiments demonstrates the benefits of our proposed model for problem-solving on multimodal sentiment analysis. The following are our primary contributions.

- We propose a CNN and Transformer-based approach for multimodal sentiment analysis that aims to extract more comprehensive text and image features. Our method uses CNN to extract local features and Transformer to capture global features, which are then combined to obtain a better representation of the data.
- We employ a supervised contrastive Learning approach with data augmentation to improve the performance of our method. By using the supervised contrastive learning loss, we encourage the embedding vectors of the same class to be closer to each other and those of different classes to be farther apart. This approach better characterizes the intra-class similarity and improves the robustness of our method.

## 2. Related Work

### 2.1. Multimodal Learning

In [20], they provided a systematic summary and future outlook for multimodal learning in 2021, kicking off the multimodal learning research boom. In [21], they proposed Category-based Deep Canonical Correlation Analysis (C-DCCA), a novel deep learning model for projecting the modal information of image and text into the same semantic space and completing the matching and retrieval of heterogeneous information.

Song et al. [22] proposed multimodal stochastic RNNs networks (MS-RNN), which extract semantic information from two different modalities and convert it into text subtitles. As a subdivision direction of multimodal learning, multimodal fusion is the basis for our work, such as multimodal sentiment analysis, so it is widely used [23,24]. The multimodal data fusion step includes four parts: single-modal feature extraction, feature fusion, model classification, and result output. According to different fusion strategies, it can be divided into early fusion, late fusion, and intermediate fusion. Early fusion is mainly achieved through dot product operation or series concatenation, and the time synchronization between multimodal features could be better. It is not easy to obtain cross-correlation between modes. Late fusion uses the corresponding models to train different modalities and then fuses the output results of these models, increasing the fusion difficulty. Intermediate fusion combines the advantages of the first two. Due to the flexibility and diversity of deep learning models, it is more suitable to use intermediate fusion methods. With the development of deep learning technology, structures, such as convolutional neural networks, and attention has been applied to intermediate fusion and achieved good results [25,26].

Currently, the most common graphic and text fusion method assumes that images and text have a one-to-one correspondence. However, in most online social scenarios, users attach multiple images to enhance the vividness of the text and fully display the emotional information. These images supplement the text and aid in the expression of emotions. As a result, this paper investigates sentiment analysis technology without sufficient cross-modal fusion feature information [27]. The image is matched with the text content using the multi-head attention mechanism and CNN, and the cross-modal information of graphics and text is deeply integrated. Furthermore, existing graphic sentiment classification methods typically only mine the association interaction information between modalities while ignoring the unique properties of models.

### 2.2. Multimodal Sentiment Analysis

Multimodal sentiment analysis is used as a combination of verbal and non-verbal features to complete user sentiment analysis. More precisely, the field aims to mine emotions, interpretations and feelings by observing people’s language, facial expressions, speech, music, movements, and more [1]. Common public datasets in this field include CMU-MOSEI [17], Memotion Analysis [28], CH-SIM [29], CMU-MOSEAS [30], B-T4SA [31], MEMOTION 2 [32], and others. Xu et al. built the Multisentinet deep semantic network to extract deep semantic features from images, which they then used to extract objects and scenes from images as additional information for multimodal sentiment analysis [9]. Multisentinet’s proposal outperforms traditional feature extraction methods in terms of the high correlation between extracted semantic features and human emotions. Many excellent models are actively produced in this field’s development, resulting in higher recognition rates and F1 values. Poria et al. presented a multimodal sentiment analysis model based on LSTM that captures contextual information in the video’s environment to aid in sentiment classification, achieving a performance improvement of more than 5% [33]. The underlying feature extraction method in text, voice, and video still has room for improvement, and feature fusion is also hierarchical, with no sequential relationship between different modes. In [34], they proposed a deep multimodal attention fusion model (DMAF) that uses hybrid fusion to leverage intrinsic correlations between visual and textual features for joint sentiment analysis, and the model achieves good results in weakly labeled and regular datasets. Truong et al. investigated the visual aspect attention network (Vistanet) in 2019, which employs attention to direct the model’s attention toward key sentence information in the document rather than visual information as a feature [35]. In [36], they proposed the attention-based heterogeneous relational model (AHRM) in 2020. The model uses a progressive dual attention module to capture the correlation between images and text and learns the image-text joint representation from the perspective of content information by combining rich social information to improve the model’s performance. In 2021, Wu et al. focused on developing a text-centric Shared Private (TCSP) framework [37]. The framework concentrates on text modalities, extracts shared and private semantic information from the other two modalities to supplement modal text information, and proposes a new method for training multimodal sentiment analysis models with unlabeled data. Tan et al. investigated multimodal emotion recognition using face and EEG, facial features extracted with CNN, final classification with soft-max, multiple voting in the late fusion layer, and the final classification results of the two modalities combined with the threshold method, as well as statistical simulation methods to obtain the final multimodal emotion classification results [38]. In [39], they proposed a social network-based real-time traffic accident monitoring framework that uses sentiment analysis technology to identify the polarity of traffic incidents through user comments, which is useful for identifying the polarity of traffic incidents and accurately understanding the situation of traffic incidents. To improve classification task accuracy, the proposed word embedding model converts formal and informal words into low-dimensional vector representations. Li et al. combine multi-layer fusion with contrastive learning and multi-modal sentiment analysis. The token-level feature fusion method, according to this method, is better suited to fusing local information in images and text than the previous feature fusion layer [40].

At present, most image-text sentiment analysis methods realize the interaction between modal features by sharing the feature vectors of the neural network representation layer, and design specific connection units to effectively integrate multiple modal features. Although the preceding literature has achieved success in terms of accuracy, issues such as a lack of training samples and the inability to effectively capture multi-level feature information in each modality remain. In the current research, we mainly solve the problems of irrelevant information interference and incomplete fusion features, so that the model can learn more emotional common features in the fusion stage after extracting the data features of each modality. Based on the models in the above literature and the analysis of the current problems, we proposed the MLFC module, and verified the effectiveness of the proposed model after continuous experiments. In order to reduce the interference of irrelevant features and learn more emotional common features, we adopt supervised contrastive learning method, so that the features of different categories are far away from each other and similar features are close to each other.

## 3. Materials and Methods

**Figure 2:**
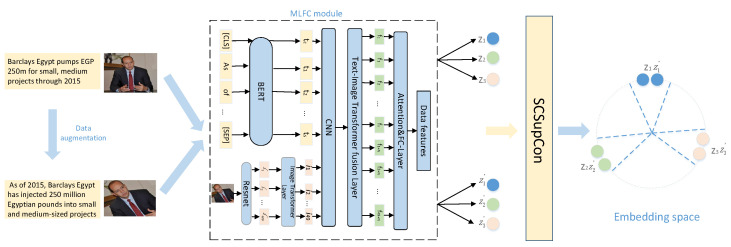
Illustration of our Model overall framework diagram. To judge sentiment polarity, the proposed architecture employs supervised contrastive learning and a CNN-connected Transformer fusion. The MLFC module is intended to solve feature extraction and fusion problems in input data. Invariance occurs for graphic sample features when embeddings from the same category, such as $z_{1}$ and $z_{1}^{\prime }$. Different categories of graphic features, such as $z_{1}$ and $z_{2}$, on the other hand, are far apart.

Using labeled multimodal data, we hope to train a feature embedding network. Embedding vectors from the same class should be close together, whereas embedding vectors from different classes should be far apart [41]. Our method adjusts the supervised classification using supervised comparative learning. Classification performs data augmentation with a batch of input data for a model. Under different data enhancement of text and image, the embedding vector of the same instance will not be changed, and the embedding vector of different instances will be different. Figure 2 shows how to enter the original graphic example and the enhanced example, enter the MLFC module and obtain the feature sequence after the fusion of graphics and text. Finally, embodied in the feature space, so that similar features are closer and different classes are far from each other, we design the SCSupCon module here. The multilayer fusion module maps the embedding vectors to the output layer. A cross-entropy loss and supervised comparative learning loss are calculated at the network’s output. In this section, we will introduce the overall framework used, monitor the comparison loss and multilayer fusion modules, and outline the optimization of the multilayer convergence approach.

### 3.1. Representation Learning Framework

Contrastive learning is a self-supervised learning method inspired by recent contrastive learning methods. Because different modal data need to pay more attention to their invariant features after fusion, SCSupConLoss maximizes the immutability of learning similar features through potential contrast loss. Emotionally related features exist in these unchanged features to ensure that the real meaning the user wants to express will not change with the text change and that the real meaning the user wants to express will not change. Figure 2 shows that the overall model framework is divided into four main components.

#### 3.1.1. Data Augmentation

Data augmentation, also known as data enrichment, is the process by which limited data yields the equivalent value of more data without significantly increasing the data. Using data augmentation improves the robustness and generalization of your model. Because the data contains text and images and different modal data must pay more attention to their unchanging characteristics after fusion, data augmentation is required.

Back-translation is an option for text data enhancement because it can generate different interpretations while preserving the semantics of the original sentence [42]. Back translation is the process of translating one sentence into another language and then back into the original language, comparing the differences between the two sentences so that the new sentence can be used as enhanced text. RandAugmentation was chosen as the data enhancement method in the image because it does not require tagging data and is more concise and convenient to sample uniformly from the same set of enhancement transformations [43]. RandAugmentation learns data augmentation strategies using a simple raster search method, and RandAugmentation can significantly reduce the incremental simple grid search by data augmentation, incorporating it into the model training process and avoiding execution as a separate preprocessing task.

#### 3.1.2. Encoder Network

The encoder network is primarily in charge of processing the encoding sequence before the fusion of input text and image generation in various processing methods. The encoder network receives a set of image and text pair data as input, and the image selection Resnet-50 model extracts features. $M_{c}^{\prime }$ is the image feature produced by Resnet’s final convolutional layer. The following equation depicts the feature sequence representation of the image $M^{\prime }$:

(1) $$M^{\prime }=flatten\left(M_{c}^{\prime }W_{m}+b_{m}\right)$$

The flatten function is a reconstruction of the first two dimensions of the vector spread into one dimension, where $M^{\prime }=\left\{M_{1}^{\prime },M_{2}^{\prime },...,M_{n_{i}}^{\prime }\right\}$. To obtain the final encoding of the image sequence features, we enter $M^{\prime }$ into the vanilla Transformer Encoder [44].

(2) $$M=\left\{M_{1},M_{2},...,M_{n_{i}}\right\}\left(M\in R^{n_{i}*d_{t}}\right)$$

To obtain the text hidden representation, we feed the text information into the BERT pre-trained model. We use the symbol *T* to represent text encoding information for ease of representation:

(3) $$T=\left\{T_{c},T_{1},T_{2},...,T_{s}\right\}\left(T\in R^{n_{t}*d_{t}}\right)$$

The original sample and the sample enhanced by data are fed into the same encoder network to produce two representation vectors. Because a sample set is made up of text and images, the enhanced sample network generates the final text encoding *T* and image sequence *M* generated by the encoder network.

#### 3.1.3. Multi-Layer Fusion Convolution Neural Network

Multi-Layer Fusion Convolution Neural Network (MLFC) is a multilayer fusion module that converts the encoder output into the final multimodal representation *R*. It is primarily used for the alignment and fusion of two modal data. CNN, a Transformer encoder, and an attention layer are all part of MLFC. Because the Transformer encoder is limited in that it can only capture long-range feature dependencies while ignoring the details of local features, the convolution operation is added before the Transformer. The convolution operation effectively extracts local features but has limitations when capturing global feature representation. As a result, combining the two can yield better results. The following is an equation:

(4) $$F=\left\{f_{1},f_{2},...,f_{n_{t}+n_{i}}\right\}=Transformer\left\{CNN\left[concat\left(T,M\right)\right]\right\}$$

To align and fuse the text and image features, we connect the text feature *T* and the image sequence feature *M*. As the text-image fusion layer, we use the new multilayer transformer encoder and CNN, which will align and fuse multi-mode capabilities. The featured result *F* of the fusion sequence of text and image can then be obtained.

Finally, the sequence features for text and image fusion are obtained, where $n_{t}+n_{i}$ is the result of graphical stitching, but the sequence features cannot be used directly for the classification task. As a result, we employ a superficial attention layer to generate a multimodal representation of R=Attention(F). Enter the fusion result *F* into Attention to obtain the final multimodal fusion representation, which is denoted by the symbol *R*.

### 3.2. Sentiment Classification Contrastive Loss

We explain how to incorporate supervised contrastive learning into sentiment classification loss.

#### 3.2.1. Supervised Contrastive Losses

Traditional contrastive learning is applied to unsupervised learning, with no label information, the same image is one class, *i* photos are twice random data augmentation, the result of two data improvements of the same image is positive samples, and other 2*i*-2 images are negative samples [45]. The loss we use is the loss of supervised comparative learning, which is to make better use of label information so that the characteristics of the same type of things are closer and the characteristics of different types of things are farther away. Supervised contrastive loss (*SupCon*) can handle the situation where multiple samples are known to belong to the same class due to the presence of labels:

(5) $$L_{SupCon}=\sum_{i=1}^{n}\frac{-1}{\left|P(i)\right|}\sum_{p\in P(i)}log\frac{exp(z_{i}\cdot z_{p}/\tau )}{\sum_{a\in A(i)}exp\left(z_{i}\cdot z_{a}/\tau \right)}$$

$L_{SupCon}$ is the loss of Supervised contrastive loss. P(i) contains the indices of positive samples in the augmented batch (original + augmentation) with respect to $z_{i}$ and |P(i)| is the cardinality of P(i). $z_{i}$ is an anchor. $z_{a}$ are negative samples. $z_{p}$ are positive samples and A(i) is the index set of negative samples.

#### 3.2.2. Sentiment Classification Loss

In the sentiment classification task, the multi-layer fusion module’s graphic-text sentiment representation *R* is transferred to the fully connected layer, and the soft-max function is used for sentiment classification. The sentiment classification loss is calculated using the cross-entropy loss function, which is widely used in deep learning:

(6) $$L_{SC}=Cross-Entropy\left(GELU\left(RW_{sc}+b_{sc}\right)\right)$$

$L_{SC}$ represents cross-entropy loss. Here the activation function selects the *GELU* function, and the $W_{sc}$ and $b_{sc}$ are hyperparameters.

#### 3.2.3. Sentiment Classification Supervised Contrastive Loss

We propose Sentiment Classification Supervised Contrastive loss (SCSupCon), which incorporates SupConLoss and SCLoss for supervised embedded learning.

(7) $$L_{SCSupCon}=\lambda_{sc}L_{sc}+\lambda_{SupCon}L_{SupCon}$$

$\lambda_{sc}$ and $\lambda_{SupCon}$ are coefficients that balance different training losses. Sentiment Classification Supervised Contrastive loss is composed by combining cross-entropy loss and supervised contrastive learning loss through two different adjustment coefficients.

SCSupCon has the following advantages:

- Samples with the same label and enhanced samples are the molecular weight of the SupConLoss formula for the same batch of original pattern pairs. The supervised contrastive learning loss mechanism stimulates the encoder to provide a closer representation of the same class so that samples of the same class are more closely combined in the embedding space [46].
- SCSupCon optimizes the separation distance in the normalized hypersphere using the angle–arc relationship. As a result, SCSupCon may show a more pronounced separation between the nearest classes on the Loss sphere’s surface.

## 4. Experiment

We built the proposed model using the Pytorch framework, with ACC and F1 as the primary evaluation indicators. Our research was conducted on a high-performance computing (HPC) node with one NVIDIA 3090 GPU and one A40 GPU. The parameters of the pre-trained BERT model and the Resnet-50 model are fixed, while the parameters of word embedding and attribute embedding are constantly updated during the training process [19]. Word and attribute embeddings were trained on the Twitter dataset using Glove [47].

For the MVSA dataset, the F1 we use is Weighted-F1 [48], while for the HFM dataset, we chose Macro-F1.

### 4.1. Data Processing

The MVSA-Single and MVSA-Multiple datasets are publicly available datasets in the field of multimodal sentiment analysis that were collected via Twitter, where users post messages with text, images, hashtags, and so on. Each text-image combination corresponds to a distinct sentiment label. Positive, neutral, and negative sentiment labels are manually applied to the MVSA dataset. The MVSA dataset is divided into two parts: MVSA-Single (MVSA-S), which contains 4869 image-text pairs labeled by one annotator with only one sentiment label, and MVSA-Multiple (MVSA-M), which contains 19,598 image-text pairs labeled by three annotators with three sentiment labels. The other component is MVSA-Multiple, where each sample is annotated by three annotators and contains three sentiment labels, totaling 19,598 image-text pairs.

We process the original two MVSA datasets in the same way as the literature [9]. We randomly divide the MVSA dataset into training sets, validation sets, and test sets using a split ratio of 8:1:1. For HFM datasets, we used the same data preprocessing method described in the literature [19], the difference we use that the datasets are randomly partitioned. The development and test sets were manually checked to ensure the accuracy of the labels. The primary method is to use the NLTK toolkit to separate words, emojis, and labels. The symbol # is used to divide the labels and replace uppercase letters with lowercase letters.

MLF is a multi-layer fusion method based on Transformer-Encoder that uses multi-head attention alignment and fusion graphic features. MLFC is a fusion method that adds convolution operations on top of MLF. The other hyperparameters are listed in the table below. On the MVSA dataset, we first trained and tested our implementation. In particular, we report the ACC and F1 values for the datasets MVSA and HFM in the evaluation. All experiments are carried out in a pre-configured environment. Table 1 shows the detailed experimental configuration. Back-translation and RandAugment are two terms that describe how text and image data are enhanced. The table also shows the specific sentiment polarity of the three datasets, and “SCSupCon” represents the loss naming of the design, which includes sentiment analysis loss and supervised versus learning loss. Section 4.3 and Section 4.4 provide experimental details.

### 4.2. Comparative Experiments

We started with MVSA-Single and then proved the model’s validity on the remaining two datasets: MVSA-Multiple and HFM. The results showed that our model reached the current comparable to the SOTA model. Our model is compared to the unimodal and multimodal baseline models. The following tables present them separately.

Text baseline model: as we can see, the image-based model performs poorly, whereas the text adoption CNN has begun to perform better, demonstrating the significance of the text model. The TGNN model is a new GNN-based model that constructs graphs with shared global parameters for each input text instead of constructing a single graph for the entire corpus. This method eliminates the dependency burden between individual texts and the entire corpus, allowing for online testing while still preserving global information [49].

On images, the Resnet and the OSDA models are chosen as the baseline models. The OSDA model is an image sentiment analysis model that focuses on both targets and scenes, and image features from various perspectives aid in the analysis of users’ sentiments [14].

Here we chose MGNNS and CLMLF as multimodal models for the MVSA dataset. The MGNNS model is a multi-channel graph neural network for image text sentiment detection. The multi-channel graph neural network learns global features of multimodal representation, and the fusion method of the model is implemented by the multi-head attention mechanism [48]. The CLMLF model is a multilayer fusion and contrast learning application that uses multilayer fusion to align and fuse token-level features of text images and two contrast. The D&R Net model is a decomposition and relational network-based model that establishes cross-modal contrast and semantic correlation at the same time (D&R network). The relationship network represents the semantic association in a cross-modal context, while the decomposition network represents the similarities and differences between images and text [50].

First, the MVSA dataset is compared with other models to evaluate the effectiveness of our model, and the experimental results are shown in the table. These are representative models based on text, images, and multimodality, arranged in order.

As shown in Table 2, the multimodal sentiment analysis model outperforms most single-modal sentiment analysis models on the MVSA dataset. Furthermore, due to the low density of image information, capturing feature information is complex, and the sentiment analysis effect on image mode is the worst. Our model outperforms the current best SOTA model on the MVSA-Single dataset, with ACC and F1 improving by 1.10% and 2.15%, respectively. The ACC effect is comparable to the MVSA-Multiple dataset, and F1 is improved by 0.52%. In general, our model is comparable to the SOTA model.

As illustrated in Table 3, our model’s performance improves by 1.21% and 1.35%, demonstrating the benefits of our proposed model in multimodal sentiment analysis. First, the proposed Multi-Layer Fusion Convolution Neural Network (MLFC) can capture the interaction between images and text at a finer level. Then, valuable complementary information can be extracted by supervising the contrast-loss sentiment classification and de-motivation encoder to provide a more accurate representation of the same kind. SCSupCon processes samples located at the decision boundary in multimodal sentiment analysis to achieve more accurate sentiment prediction.

### 4.3. Ablation Experiments

We conducted ablation experiments on the three datasets to demonstrate the effectiveness of supervised contrast learning and MLFC, and the results are shown in Table 4.

Table 4 shows that the model’s performance decreases when the contrast learning approach and the MLF module are used. When the experimental ablation results Sentiment Classification Supervised Contrastive loss (SCSupCon) is added, the contrast learning effect is enhanced. The final experimental results show that combining the two modules makes the model more valuable, and combining SCSupCon with Multi-Layer Fusion Convolution Neural Network (MLFC) helps to obtain local features and improve model performance in general. All of the experimental metrics have improved, demonstrating the model’s effectiveness.

### 4.4. Data Visualization

**Figure 3:**
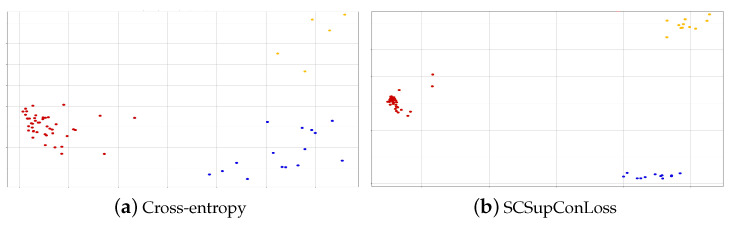
Loss function clustering visualization. In supervised comparative learning, clustering can be accomplished by embedding samples into low-dimensional embedding spaces and then using clustering algorithms to divide the samples in the embedding space into different clusters. The red, blue, and yellow dots in the embedding space represent the aggregation of different classes of samples, and we can see how our model loss function outperforms the cross-entropy loss function.

To test whether our supervised comparative learning task can assist the model in learning common features related to sentiment in multimodal data, we run feature space visualization experiments on the dataset. By reducing dimensionality, visualize the data feature vectors at the model’s final layer. Figure 3a depicts the visualization of cross-entropy loss using the model, and Figure 3b depicts the visualization of the model’s results using SCSupConLoss. The figure shows that adding supervised comparative learning increases the distance between positive and negative emotions in the vector space and increases the degree of data aggregation. This demonstrates that the model distinguishes these data in vector spaces based on shared features in the sentiment data. This demonstrates that the model distinguishes these data in vector spaces based on shared features in the sentiment data. Because the amount of neutral sentiment data is relatively small, our model’s visualization aggregates the neutral data rather than scattering it across the vector space as if it were only cross-entropy losses [51]. All of this suggests that incorporating supervised contrastive learning can help the model learn common features associated with emotions more effectively, thereby improving the model’s performance.

**Figure 4:**
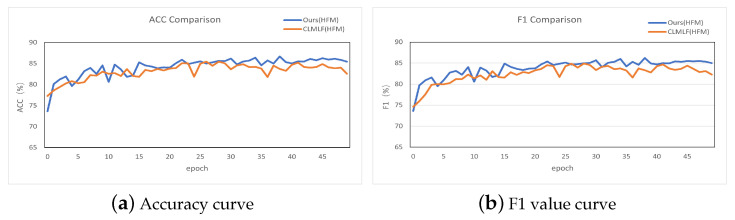
Accuracy and F1 value plot.

Figure 4 depicts the accuracy and F1 value curves of our model and CLMLF on the HMM test set after 50 epochs of training. As shown in Figure 4a, our model has a shorter growth curve and higher accuracy than CLMLF in terms of accuracy as a percentage of the total sample of correctly predicted results. In comparison to CLMLF, our proposed model’s performance fluctuates less and gradually stabilizes after 40 epochs. F1 Score is a statistical measure of classification model accuracy that is a harmonic average of model accuracy and recall. Figure 4b shows that our model achieves an F1 value greater than the CLMLF of 1.35% and stabilizes after 40 epochs, resulting in better classification results.

### 4.5. Case Study

Here are a few illustrative examples to help you understand the validity of our model more intuitively. A case study is given here to help demonstrate the effectiveness of the model we designed. We compared sentiment labels based on our model and BERT predictions.

The leftmost column in the case study is the example image, the second column is the text information corresponding to the image, the third column is the predicted sentiment result of the BERT pre-trained model, and the last column is our model performance, which is useful for comparison with the BERT pre-training model.

As shown in Table 5, we can find that if we only consider the emotion of the text in the sentiment analysis task, it is not easy to analyze the user’s emotional tendency correctly. For example, the first data in Table 5 has a negative meaning, but the smiley face has a positive meaning when combined with the image expression. If we only look at the text for the second data, we can see that it may express a neutral meaning. When we add an image, we discover that it is a negative sentiment image expressing a negative emotion. The case study in this section demonstrates how well our model captures multimodal information and interactions.

## 5. Conclusions

We propose a multimodal sentiment analysis model based on supervised contrastive learning to improve the performance of multimodal sentiment analysis. Our model addresses the problems of insufficient multimodal fusion feature information and fusion feature interference information by adopting supervised contrastive learning and a multi-layer fusion module. Our model combines CNN and a multi-layer fusion module to obtain local features, which are then stitched together to avoid interference from irrelevant information in the sentiment analysis task. This helps us more effectively obtain emotional features. We also use the Transformer in our model to obtain global features, improving the fusion effect and obtaining more understandable graphic features. By using supervised contrastive learning and data augmentation, we reduce the interference of irrelevant information and highlight valuable features. Our proposed model achieves good detection performance on the MVSA and HFM datasets, improving the Acc and F1 evaluation indicators.

In the future, to solve the feature redundancy problem for sentiment analysis tasks, the feature extraction method that is better for multimodal data is considered in the existing encoder network. Additionally, we will investigate new multimodal fusion methods and consider adding audio modality to solve the problem of incomplete cross-modal feature fusion.

## Figures and Tables

**Table 1:** Hyperparameter setting display.

Hyperparameter	MVSA-Single	MVSA-Multiple	HFM
Text data augmentation	back-translation	back-translation	back-translation
Image data augmentation	RandAugment	RandAugment	RandAugment
Emotional polarity	3	3	2
Loss	Conloss/SCSupConloss	Conloss/SCSupConloss	Conloss/SCSupConloss
Contrasting learning styles	Self-Supervised/Supervised	Self-Supervised/Supervised	Self-Supervised/Supervised
Text Encoder	BERT	BERT	BERT
Image Encoder	Resnet-50	Resnet-50	Resnet-50
Fuse model epoch	20	20	20
Epoch	30	30	30
Integration method	MLF/MLFC	MLF/MLFC	MLF/MLFC
Optimizer	Adams	Adams	Adams
Learning rate	2 × 10${}^{-5}$	2 × 10${}^{-5}$	2 × 10${}^{-5}$
Batch	32/32	64/64	128/48

**Table 2:** Using the MVSA dataset and HFM set, the results of the results show the ACC and F1 performance (%) of our designed model, and our experimental results are highlighted in bold.

Modality	Model	MVSA-Single		MVSA-Multiple	
		Acc (%)	F1 (%)	Acc (%)	F1 (%)
Text	CNN	68.19	55.90	65.64	57.66
	BiLSTM	70.12	65.06	67.90	67.90
	BERT	71.11	69.70	67.59	66.24
	TGNN	70.34	65.94	69.67	61.80
Image	ResNet-50	64.67	61.55	61.88	60.98
	OSDA	66.75	66.51	66.62	66.23
Multimodal	MultiSentiNet	69.84	69.84	68.86	68.11
	HSAN	69.88	66.90	67.96	67.76
	Co-MN-Hop6	70.51	70.01	68.92	68.83
	MGNNS	73.77	72.70	N/A	–
	CLMLF	75.33	73.46	70.53	67.45
	Ours	**76.44**	**75.61**	**70.53**	**67.97**

**Table 3:** For the HFM set, the results in the table show the ACC and F1 performance (%) of our design model evaluation metrics. Our experimental results are highlighted in bold.

Modality	Model	HFM	
		Acc (%)	F1 (%)
Text	CNN	80.03	75.32
	BiLSTM	81.90	77.53
	BERT	83.89	83.26
Image	ResNet-50	72.77	71.38
	ResNet-101	72.48	71.22
Multimodal	Concat (2)	81.03	77.99
	Concat (3)	81.74	78.74
	MMSD	83.44	80.81
	D&R Net	85.02	80.60
	CLMLF	85.43	84.87
	Ours	**86.64**	**86.22**

**Table 4:** Ablation study results. The results of our experiments are highlighted in bold.

Model	MVSA-Single		MVSA-Multiple		HFM	
	Acc (%)	F1 (%)	Acc (%)	F1 (%)	Acc (%)	F1 (%)
BERT	71.11	69.70	67.59	66.24	83.89	83.26
ResNet-50	64.67	61.55	61.88	60.98	72.77	71.38
BERT + ResNet-50 + MLF + DBCL + LBCL	75.33	73.46	70.53	67.45	85.43	84.87
BERT + ResNet-50 + MLF + SCSupConLoss	75.33	75.75	69.88	67.14	86.27	85.70
BERT + ResNet-50 + MLFC + LBCL + SCSupConLoss	**76.44**	**75.61**	**70.53**	**67.97**	**86.64**	**86.22**

**Table 5:** Example of misclassified by BERT and correctly classified by Ours.

Image	Text	BERT	Ours
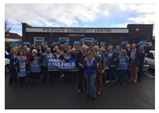	Campaigning in Polegate this morning with the ebullient MariaCaulfield and the frugal Francis Maude	Neutral	Positive
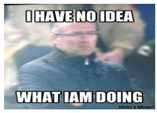	AVFCBlog oldmansaid JackWoodwardAV StanCollymore avfcforums worried avfc utv	Neutral	Negative
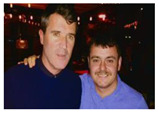	laurencekinlan: Met my hero in Cork last night, my bleeding Ronnie looks disgraceful though?	Negative	Positive

## Data Availability

Not applicable.
